# Complete Genome Sequence of the *Treponema pallidum* subsp. *pallidum* Sea81-4 Strain

**DOI:** 10.1128/genomeA.00333-14

**Published:** 2014-04-17

**Authors:** Lorenzo Giacani, Stefanie L. Iverson-Cabral, Jordon C. K. King, Barbara J. Molini, Sheila A. Lukehart, Arturo Centurion-Lara

**Affiliations:** a Department of Medicine, University of Washington, Seattle, Washington, USA; b Department of Global Health, University of Washington, Seattle, Washington, USA

## Abstract

Using the rabbit model of syphilis, the Sea81-4 strain of *Treponema pallidum* subsp. *pallidum* has been found to be more likely than other strains to invade the central nervous system (CNS). To identify possible explanations for this important phenotype at the genomic level, we sequenced the Sea81-4 strain genome.

## GENOME ANNOUNCEMENT

With ≥25 million adults affected worldwide (1), syphilis represents a significant burden in global health. Syphilis infection results in an invasion of the central nervous system (CNS) in >40% of patients (2). While some patients will spontaneously clear the invading organisms, others will develop transient or persistent inflammation, which can lead to asymptomatic neurosyphilis, meningitis, meningovasculitis, general paresis, tabes dorsalis, otologic syphilis, or ocular syphilis (3, 4). Using the rabbit model for CNS invasion and infection, the Sea81-4 strain of *Treponema pallidum* subsp. *pallidum* was found to cause persistent infection of the CNS following intravenous inoculation, unlike five other syphilis isolates (5). Furthermore, skin lesions that developed in Sea81-4-infected rabbits were shown to be less severe than those presenting in rabbits infected with other *T. pallidum* subsp. *pallidum* isolates (5). We therefore sequenced the Sea81-4 strain genome to identify possible explanations for these phenotypes at the genomic level, and also to shed further light on the subject of genetic variability and phenotype differences among syphilis strains, an underinvestigated topic in the field of *T. pallidum* subsp. *pallidum* biology that warrants exploration (5–7).

Genome sequencing of the Sea81-4 strain, originally isolated from a patient’s primary chancre in Seattle in 1980, was performed by the 454 (Roche) sequencing technology. Genomic DNA for the Sea81-4 strain was extracted following strain propagation in New Zealand White rabbits. All procedures involving laboratory animals were approved by the University of Washington Institution of Animal Care and Use Committee (IACUC). Both library construction and high-throughput sequencing were performed at the Genomics Core Laboratory of the Washington State University (WSU) School of Molecular Biosciences. Assembly of the 454 reads, performed using Newbler version 2.5.3 at the WSU sequencing facility, yielded an approximate 20-fold coverage of the Sea81-4 genome. The *T. pallidum* subsp. *pallidum* Chicago strain genome sequence (8) (GenBank accession no. CP001752.1) was used for the reference-guided assembly. Gaps between the 12 contigs obtained from the assembly were closed using PCR and Sanger sequencing. Annotation of the genome was performed by the NCBI Prokaryotic Genomes Automatic Annotation Pipeline (PGAAP) (http://www.ncbi.nlm.nih.gov/genomes/static/Pipeline.html), which utilizes GeneMark, Glimmer, and tRNAscan-SE searches.

The Sea81-4 genome is 1,139,203 bases long and contains 1,062 open reading frames (ORFs), 6 rRNA genes, and 45 tRNAs. Because the Sea81-4 strain *tprK* gene is hypervariable, a unique sequence from the seven variable (V) regions of this gene was not obtained. Therefore, in the deposited sequence, the *tprK* V1 to V7 regions are replaced by Ns. Ongoing comparative genomics analyses may allow us to understand the genetic basis of the enhanced ability of Sea81-4 to invade the CNS and cause attenuated skin lesions in the rabbit model of syphilis.

### Nucleotide sequence accession number.

The complete genome sequence of *T. pallidum* subsp. *pallidum* Sea81-4 was deposited into the GenBank database and assigned accession no. CP003679.
